# Supplementary material for: Multi‐dataset identification of innovative feature genes and molecular mechanisms in keratoconus
Source: J Cell Mol Med. 2024 Sep 19;28(18):e70079. doi: 10.1111/jcmm.70079 (PMC11412914; doi:10.1111/jcmm.70079)
Supplement: Supplementary file 2 — Material S2. [file JCMM-28-e70079-s002.zip › 7.GSEA analysis/3.DBNDD1/KEGG_STEROID_HORMONE_BIOSYNTHESIS.html]

Details for gene set KEGG\_STEROID\_HORMONE\_BIOSYNTHESIS[GSEA]

|  || Dataset | DBNDD1.group.cls#h\_versus\_l |
| Phenotype | group.cls#h\_versus\_l |
| Upregulated in class | l |
| GeneSet | KEGG\_STEROID\_HORMONE\_BIOSYNTHESIS |
| Enrichment Score (ES) | -0.40556812 |
| Normalized Enrichment Score (NES) | -1.2385548 |
| Nominal p-value | 0.115913555 |
| FDR q-value | 0.70244646 |
| FWER p-Value | 0.982 |
Table: GSEA Results Summary

  

Fig 1: Enrichment plot: KEGG\_STEROID\_HORMONE\_BIOSYNTHESIS      
 Profile of the Running ES Score & Positions of GeneSet Members on the Rank Ordered List

  

| SYMBOL | TITLE | RANK IN GENE LIST | RANK METRIC SCORE | RUNNING ES | CORE ENRICHMENT || 1 | UGT1A8 | na | 313 | 0.502 | 0.0482 | No |
| 2 | UGT2B11 | na | 1393 | 0.345 | 0.0563 | No |
| 3 | UGT2B4 | na | 2640 | 0.266 | 0.0507 | No |
| 4 | UGT1A6 | na | 2660 | 0.265 | 0.0804 | No |
| 5 | HSD3B1 | na | 3889 | 0.217 | 0.0696 | No |
| 6 | HSD3B2 | na | 4106 | 0.210 | 0.0873 | No |
| 7 | CYP11B2 | na | 4695 | 0.190 | 0.0920 | No |
| 8 | CYP7A1 | na | 5268 | 0.171 | 0.0950 | No |
| 9 | CYP17A1 | na | 5374 | 0.168 | 0.1112 | No |
| 10 | UGT2B28 | na | 6027 | 0.150 | 0.1094 | No |
| 11 | SULT2B1 | na | 6922 | 0.126 | 0.0979 | No |
| 12 | CYP3A4 | na | 8583 | 0.093 | 0.0607 | No |
| 13 | HSD17B12 | na | 9708 | 0.075 | 0.0368 | No |
| 14 | SRD5A1 | na | 9733 | 0.075 | 0.0447 | No |
| 15 | HSD17B1 | na | 10398 | 0.066 | 0.0330 | No |
| 16 | HSD17B8 | na | 12712 | 0.041 | -0.0290 | No |
| 17 | UGT2A3 | na | 13213 | 0.036 | -0.0393 | No |
| 18 | COMT | na | 15365 | 0.020 | -0.0990 | No |
| 19 | CYP11B1 | na | 16642 | 0.012 | -0.1345 | No |
| 20 | UGT2B10 | na | 17872 | 0.004 | -0.1695 | No |
| 21 | UGT2B15 | na | 18962 | -0.004 | -0.2004 | No |
| 22 | STS | na | 19953 | -0.013 | -0.2276 | No |
| 23 | UGT2B7 | na | 24690 | -0.066 | -0.3566 | No |
| 24 | HSD17B7 | na | 24763 | -0.067 | -0.3510 | No |
| 25 | CYP21A2 | na | 26060 | -0.089 | -0.3782 | No |
| 26 | CYP3A7 | na | 26069 | -0.089 | -0.3684 | No |
| 27 | CYP7B1 | na | 26304 | -0.094 | -0.3644 | No |
| 28 | AKR1C4 | na | 26497 | -0.098 | -0.3588 | No |
| 29 | CYP11A1 | na | 26903 | -0.106 | -0.3585 | No |
| 30 | SRD5A3 | na | 27378 | -0.116 | -0.3589 | No |
| 31 | UGT2A1 | na | 28049 | -0.132 | -0.3632 | No |
| 32 | AKR1C1 | na | 28597 | -0.146 | -0.3623 | No |
| 33 | AKR1D1 | na | 29423 | -0.170 | -0.3668 | No |
| 34 | HSD17B6 | na | 30768 | -0.213 | -0.3813 | Yes |
| 35 | HSD17B2 | na | 31065 | -0.223 | -0.3645 | Yes |
| 36 | AKR1C3 | na | 31537 | -0.242 | -0.3506 | Yes |
| 37 | CYP3A43 | na | 31748 | -0.251 | -0.3280 | Yes |
| 38 | CYP3A5 | na | 31787 | -0.253 | -0.3003 | Yes |
| 39 | HSD11B1 | na | 32457 | -0.291 | -0.2865 | Yes |
| 40 | HSD11B2 | na | 32724 | -0.308 | -0.2591 | Yes |
| 41 | SRD5A2 | na | 32869 | -0.319 | -0.2270 | Yes |
| 42 | CYP1B1 | na | 33997 | -0.429 | -0.2106 | Yes |
| 43 | CYP19A1 | na | 34057 | -0.437 | -0.1625 | Yes |
| 44 | SULT1E1 | na | 34158 | -0.453 | -0.1138 | Yes |
| 45 | CYP1A1 | na | 34212 | -0.466 | -0.0623 | Yes |
| 46 | HSD17B3 | na | 34651 | -0.678 | 0.0022 | Yes |
Table: GSEA details [plain text format]

  

Fig 2: KEGG\_STEROID\_HORMONE\_BIOSYNTHESIS      
 Blue-Pink O' Gram in the Space of the Analyzed GeneSet

  

Fig 3: KEGG\_STEROID\_HORMONE\_BIOSYNTHESIS: Random ES distribution      
 Gene set null distribution of ES for **KEGG\_STEROID\_HORMONE\_BIOSYNTHESIS**
